# An improved de novo genome assembly of the common marmoset genome yields improved contiguity and increased mapping rates of sequence data

**DOI:** 10.1186/s12864-020-6657-2

**Published:** 2020-04-02

**Authors:** Vasanthan Jayakumar, Hiromi Ishii, Misato Seki, Wakako Kumita, Takashi Inoue, Sumitaka Hase, Kengo Sato, Hideyuki Okano, Erika Sasaki, Yasubumi Sakakibara

**Affiliations:** 1 Department of Biosciences and Informatics, Keio University, Yokohama, Kanagawa 223-8522 Japan; 2 Department of Marmoset Biology and Medicine, Central Institute for Experimental Animals, Kawasaki, Kanagawa 210-0821 Japan; 3 Department of Physiology, Keio University School of Medicine, Shinjuku, Tokyo, 160-8582 Japan; 4 Laboratory for Marmoset Neural Architecture, RIKEN Center for Brain Science, Wako-shi, Saitama, 351-0198 Japan

**Keywords:** Common marmoset, *Callithrix jacchus*, De novo assembly, Non-human primate genomics, Chromosome-scale scaffolds

## Abstract

**Background:**

The common marmoset (*Callithrix jacchus*) is one of the most studied primate model organisms. However, the marmoset genomes available in the public databases are highly fragmented and filled with sequence gaps, hindering research advances related to marmoset genomics and transcriptomics.

**Results:**

Here we utilize single-molecule, long-read sequence data to improve and update the existing genome assembly and report a near-complete genome of the common marmoset. The assembly is of 2.79 Gb size, with a contig N50 length of 6.37 Mb and a chromosomal scaffold N50 length of 143.91 Mb, representing the most contiguous and high-quality marmoset genome up to date. Approximately 90% of the assembled genome was represented in contigs longer than 1 Mb, with approximately 104-fold improvement in contiguity over the previously published marmoset genome. More than 98% of the gaps from the previously published genomes were filled successfully, which improved the mapping rates of genomic and transcriptomic data on to the assembled genome.

**Conclusions:**

Altogether the updated, high-quality common marmoset genome assembly provide improvements at various levels over the previous versions of the marmoset genome assemblies. This will allow researchers working on primate genomics to apply the genome more efficiently for their genomic and transcriptomic sequence data.

## Background

The common marmoset (*Callithrix jacchus*) is a small, new-world monkey, which can be handled in laboratories with relative ease [1–3]. Mouse is a widely used model animal, however the genetic, physiological, and anatomical differences between mice and primates prevent their application for human studies, thus insisting on the necessity of non-human primate (NHP) models [1, 2]. Common marmosets have an effective breeding capacity relatively among primates, and they show some characteristics which are more related to humans than the other NHPs [2]. Common marmosets have been utilized as models for numerous neurological diseases [2], and a multiscale brain atlas project called Brain/MINDS had been initiated with a 10-year roadmap [4]. In addition, marmosets were the first transgenic NHPs to be generated with germline transmission [1], while also their embryonic stem cell lines and induced pluripotent stem cell lines are being widely researched [5, 6]. In light of the increasing importance of marmosets as an alternative NHP model animal for biomedical and neuroscience research, the genome was first sequenced by the marmoset genome sequencing and analysis consortium [7]. The 2.26 Gb assembled genome from a female marmoset, although was sorted out into chromosomes, contained many shorter contigs and also 187,214 gap regions. These hard to assemble gap regions cannot be ignored, as they can lead to false positive results [8], and the gap regions could harbor many functionally relevant genes [9]. Recent studies have uncovered that many genes were wrongly labelled as missing in bird genomes, because of the locality of those genes being GC-rich and hence had posed challenges in identifying them [9]. To improve such poorly assembled regions of the marmoset genome, second-generation sequencing technology (Illumina) based short reads were employed, which helped fill approximately one-third (65,384) of the gap regions [10]. However, the genome still remains largely fragmented (contig N50 = 61 kbp) aside from the numerous undetermined bps. Sequence gaps and fragmented contigs are characteristic features in genome assemblies, however, with the rise of third-generation sequencing technologies such as Pacific Biosciences (PacBio) and Oxford Nanopore sequencing technologies, a large number of genomes are being updated to high contiguity genomes with the help of longer reads [11, 12]. As an example, the genomes of apes assembled using first and second-generation sequencing data were abounded with tens to hundreds of thousands of gaps impacting multi-genome sequence alignments which limited sequence based discoveries [13]. Recently, the ape genomes of Gorilla, Orangutan, and, Chimpanzee, were sequenced and reassembled using PacBio reads resulting in large scale improvements in the respective genomes [13]. Similarly, for the common marmoset, after first- and second-generation sequencing technologies left the genome fragmented with many gaps, we have employed PacBio sequencing, a third-generation, single-molecule sequencing technology, and here, we report the updated version of the common marmoset genome with fewer gaps and high contiguity.

## Results and discussions

### De novo assembly and pseudo-chromosome construction

**Fig. 1:**
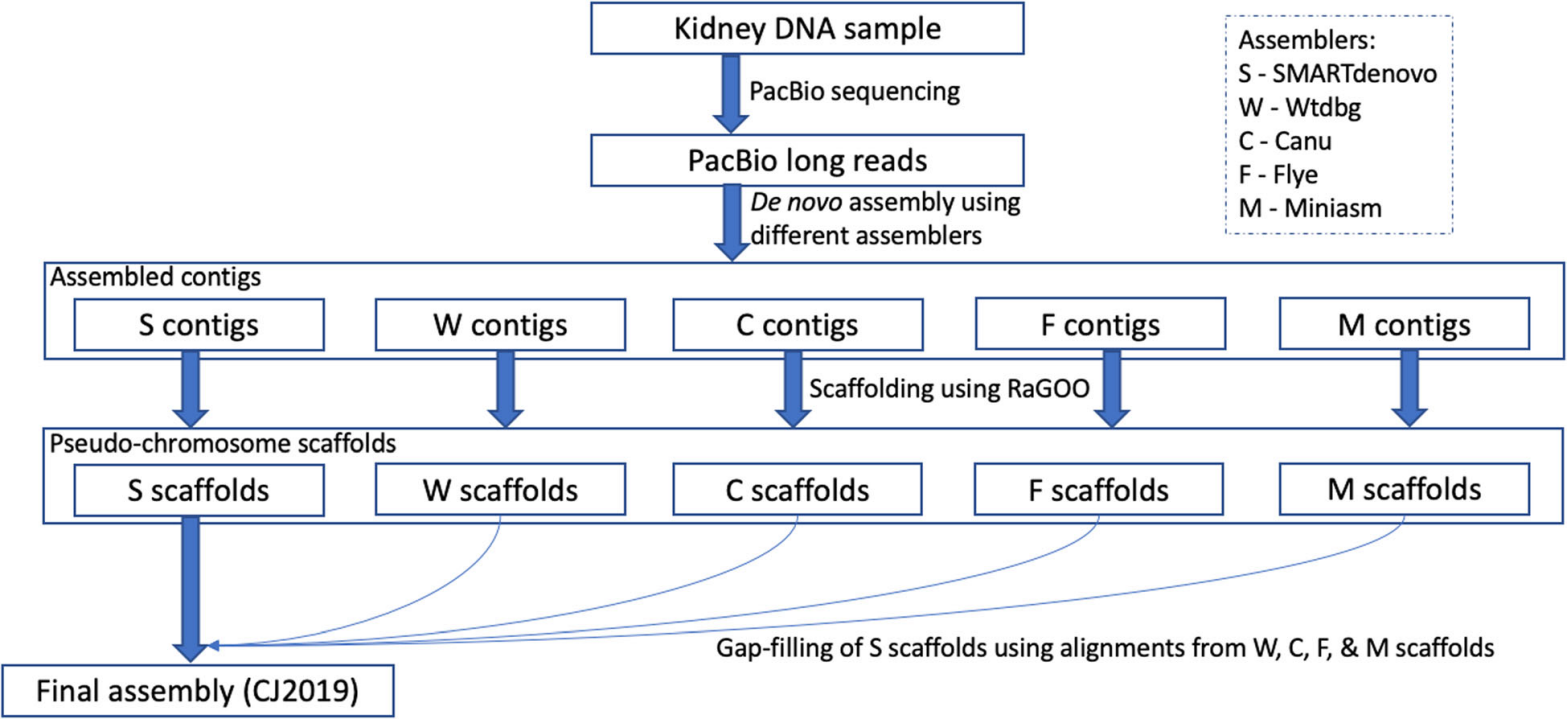
The de novo assembly workflow

The long-read sequence data obtained from the PacBio RSII sequencer amounted to 114.80 Gb, covering approximately 43 × of the genome (Fig. S1). In the context of the size of the genome, the lengths of the PacBio reads (N50: 16.41 kbp; average sequence length: 11 kbp) were only slightly shorter than the contiguous gap-free regions from the previous marmoset genome assembly (N50: 61 kbp; average sequence length: 24 kbp). After assembling the sequence data with several assembly tools as part of the assembly workflow (Fig. 1), SMARTdenovo and wtdbg assemblies produced better results compared to the other assemblers in terms of contiguity, with N50 values reaching more than 6 Mb (Table S1). SMARTdenovo produced a slightly shorter N50 in comparison to wtdbg, but was chosen as the final assembly considering that SMARTdenovo had relatively longer average contig lengths and the least number of contigs. After constructing pseudo-chromosomes for all the assemblies (Table S2), the alternate assemblies were used to fill the gaps in the SMARTdenovo pseudo-chromosomes, resulting in 1771 sequence gaps. The final assembly size was 2.79 Gb, with a scaffold N50 value of 143.89 Mb corresponding to the chromosome lengths, and a contig N50 value of 6.38 Mb (Table 1). The obtained contig N50 value compared favorably against the recently updated genome assemblies using PacBio reads, whose average contig N50 was 6.34 Mb (Table 2). The N50 values were better for the other genome assemblies, only when multiple additional technologies such as Hi-C, CHICAGO, and BioNano optical maps were employed. When considering assemblies which used only third-generation sequence data for updating the genome, the marmoset genome assembly’s contig N50 ranked the best among them, in spite of the relatively longer genome size (Table 2). The assembly was estimated to comprise 39.37% repeat content. As expected in the primate genomes, the LINE1 elements contributed to most (21.44%) of the repeats. SINES (9.18%) and LTR elements (5.24%) were also distributed throughout the genome (Table S3). Only a very small percentage (0.07%) of the repeats was left unclassified. A total of 18,385 gene models, along with 78,992 alternatively spliced transcripts, were obtained using the combined approach.

**Table 1:** The common marmoset genome assembly statistics

	Contigs	Scaffolds
# sequences	1788	65
Total assembled sequence	2.79 Gb	2.79 Gb
Longest sequence length	46.03 Mb	213.27 Mb
N50	6.38 Mb	143.89 Mb
N75	2.58 Mb	115.91 Mb
L50	117	8
L75	289	14

**Table 2:** A survey of contig N50s obtained in recent studies, involving improvement of genomes, primarily using long read sequence technologies

Species	Common name	Assembled genome size	Contig N50	Additional technologies*
*Aedes aegypti*	Mosquito	1.28 Gb	11.76 Mb [11]	Yes
*Brassica rapa*	Brassica	0.35 Gb	1.44 Mb [14]	Yes
*Bubalus bubalis*	Water buffalo	2.6 Gb	22.4 Mb [15]	Yes
*Calypte anna*	Anna’s hummingbird	1.01 Gb	5.36 Mb [16]	No
*Camponotus floridanus*	Ant	0.28 Gb	1.22 Mb [17]	Yes
*Capra hircus*	Domestic goat	2.92 Gb	18.7 Mb [18]	Yes
*Columba livia*	Rock pigeon	1.10 Gb	0.02 Mb [19]	Yes
*Fragaria vesca*	Woodland strawberry	0.22 Gb	7.9 Mb [20]	Yes
*Gallus gallus*	Chicken	1.21 Gb	2.9 Mb [21]	Yes
*Gorilla gorilla*	Gorilla	3.08 Gb	10.02 Mb [22]	Yes
*Harpegnathos saltator*	Ant	0.34 Gb	0.88 Mb [17]	Yes
*Hordeum vulgare* L. var. nudum	Tibetan hulless barley	4.00 Gb	1.56 Mb [23]	Yes
*Pan troglodytes*	Chimpanzee	2.99 Gb	12.42 Mb [13]	Yes
*Pongo abelii*	Orangutan	3.04 Gb	11.07 Mb [13]	Yes
*Rubus occidentalis*	Black raspberry	0.29 Gb	5.1 Mb [24]	Yes
*Siraitia grosvenorii*	Monk fruit	0.46 Gb	0.43 Mb [25]	No
*Symphodus melops*	Corkwing wrasse	0.61 Gb	0.46 Mb [26]	No
*Taeniopygia guttata*	Zebra finch	1.14 Gb	5.80 Mb [16]	No
*Zea mays*	Maize	2.10 Gb	1.18 Mb [12]	Yes

*Additional technologies include Hi-C, CHICAGO, BioNano optical maps, and others

### Evaluation of the assembly

As of March 31, 2019, the NCBI database for assembly of the common marmoset genome contained four assemblies: i) the first genome submitted at 2010 [7], ii) the improved genome using Illumina submitted at 2015 [10], iii) an Ion-torrent based genome submitted at 2015, and iv) an Illumina based genome anchored by Hi-C and CHICAGO libraries submitted at 2017 (Table S4). The ion-torrent based assembly contained more than one Gb of the genome missing and hence was ignored for the evaluation purpose, while the rest of the assemblies were designated names in this manuscript, according to their submission years as CJ2010, CJ2015, and CJ2017, respectively. The genome assembly presented in this study was designated as CJ2019. When BUSCO [27] was executed for the assemblies, the BUSCO scores yielded 92.9% completeness for both CJ2017 and CJ2019 assemblies, and 92.1 and 91.9% completeness for CJ2010 and CJ2015 assemblies respectively. This indicated that all the assemblies had high level of completeness at the level of conserved genes. The major differences between the current assembly and the previous assemblies were observed in contiguity, number of sequence gaps, and mapping rates of sequence data.

### Improved contiguity

**Fig. 2:**
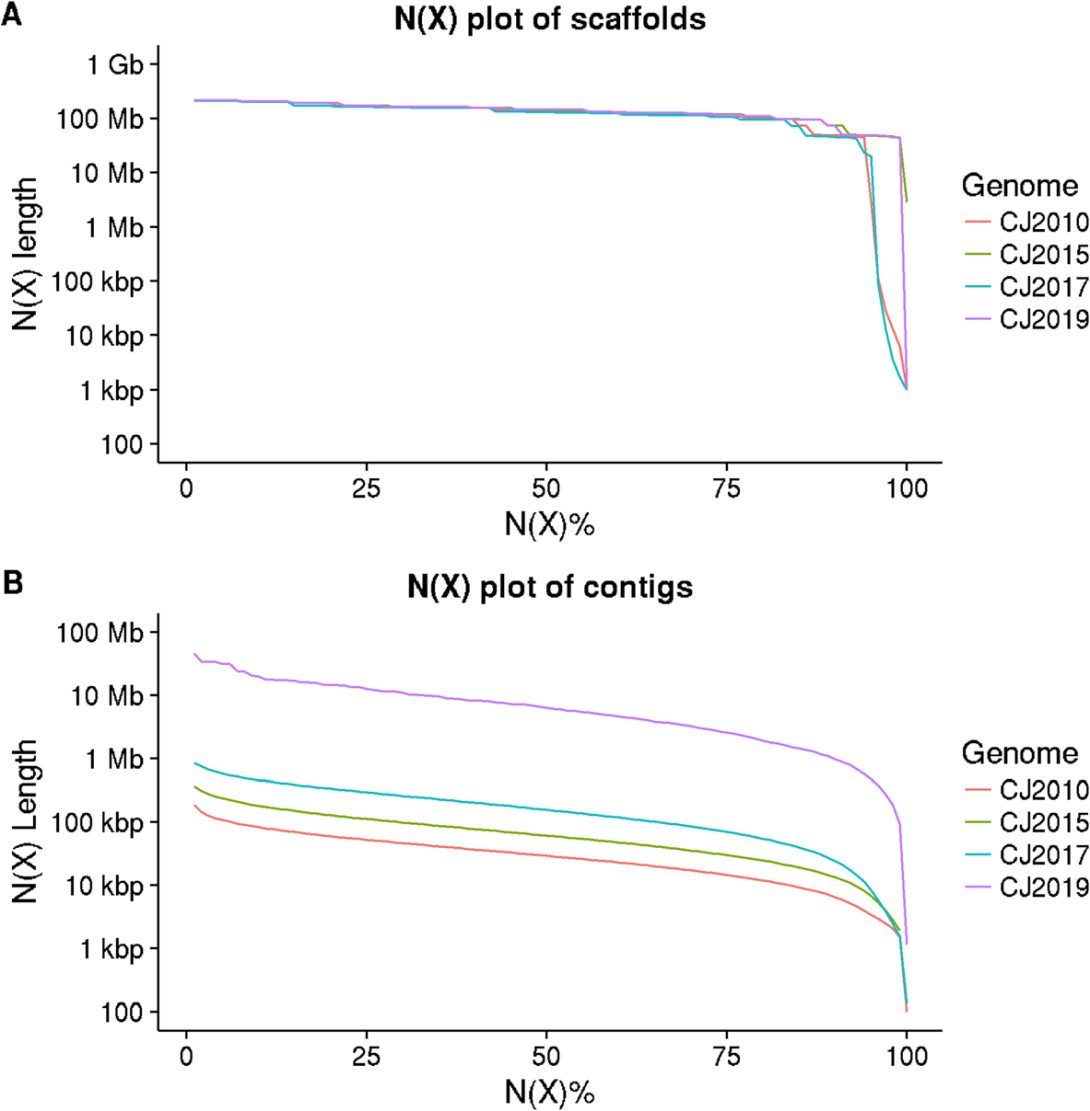
Contiguity of all the marmoset genome assemblies (CJ2010, CJ2015, CJ2017, and CJ2019). **a**) N(X) plot of scaffolds, **b**) N(X) plot of contigs

Using the contig N50 value as a metric, CJ2019 produced 217.76, 104.42, and 41.06 fold contiguity improvements over CJ2010, CJ2015, and CJ2017 assemblies, respectively (Fig. S2). The CJ2019 assembly also showed a range of 36.88 to 177.22 fold improvements in contig N75 values over the previous marmoset assemblies (Fig. S2). To further insist on the quality of the CJ2019 assembly at the contiguity level, 2.50 Gb of the 2.79 Gb genome was represented in contigs which were longer than 1 Mb. Also, 54 contigs were of length more than 10 Mb, while it should be noted that there was not even a single contig which managed to reach a length of 1 Mb in any of the previous marmoset assemblies. When the N(x) values are plotted, although the scaffold N(x) values were similar across the assemblies (Fig. 2a), the other assemblies fell below in comparison to the CJ2019 contig graph (Fig. 2b). All the assemblies produced scaffolds in the range of chromosomal lengths, with the N50 values reaching 132.17 Mb, 140.45 Mb, 129.2 Mb, and 143.89 Mb for CJ2010, CJ2015, CJ2017, and CJ2019 assemblies respectively.

### Reduced sequence gaps

The other major improvement in the CJ2019 assembly is a significant reduction in the number of sequence gaps (Table 3). While the CJ2015 genome closed approximately 41% of the gaps in the CJ2010 genome, the current CJ2019 genome resulted in 1771 gaps, closing more than 98% of the sequence gaps from the previous versions of the marmoset genome. This resulted in more than 99% of the genome being sequenced, leaving only complex regions such as centromeric repeats, and large segmental duplications to be filled. The closing of the gaps greatly facilitated the mapping of all kinds of data onto the CJ2019 assembly, increasing the space for repeat and gene identifications.

**Table 3:** Statistics of the gaps in the published marmoset genome assemblies

	# Gaps			Improvements in CJ2019	
Chromosome	CJ2010	CJ2015	CJ2019	Vs CJ2010	Vs CJ2015
1	13,245	7859	177	98.66	97.75
2	11,874	6677	84	99.29	98.74
3	9897	5404	48	99.52	99.11
4	9532	5271	83	99.13	98.43
5	11,928	7264	166	98.61	97.71
6	8890	4993	116	98.70	97.68
7	10,453	6137	64	99.39	98.96
8	7048	3956	26	99.63	99.34
9	8252	4781	66	99.20	98.62
10	8328	4673	57	99.32	98.78
11	8419	5131	113	98.66	97.80
12	8444	5023	72	99.15	98.57
13	6469	3549	45	99.30	98.73
14	6444	3702	75	98.84	97.97
15	5580	3193	36	99.35	98.87
16	5409	3114	24	99.56	99.23
17	3879	2086	13	99.66	99.38
18	3031	1762	39	98.71	97.79
19	3128	1861	31	99.01	98.33
20	3159	1899	32	98.99	98.31
21	2872	1713	25	99.13	98.54
22	6638	4783	67	98.99	98.60
X	10,542	7314	300	97.15	95.90
Y	269	184	12	95.54	93.48

### Improved mapping rates

**Fig. 3:**
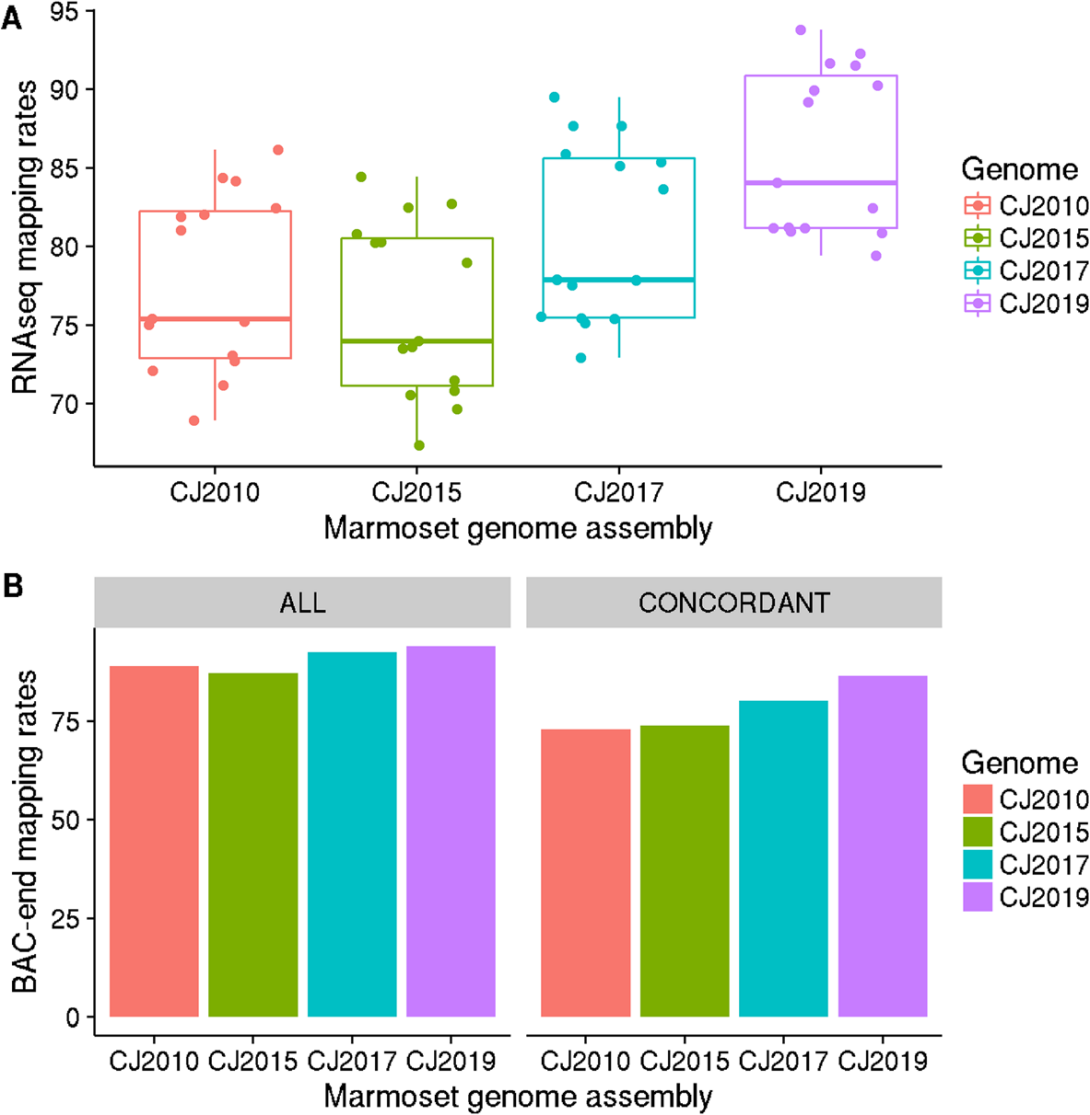
Improved mapping rates of all the marmoset genome assemblies (CJ2010, CJ2015, CJ2017, and CJ2019). **a**) Box plot of the mapping rates of RNAseq data from different brain samples, **b**) Bar plot of the mapping rates of the common marmoset’s BAC-end data

When marmoset RNAseq reads from 12 different brain tissues were aligned against the assemblies, the average mapping rate was below 80% for all the previous assemblies. In contrast, the CJ2019 genome assembly displayed more than 80% alignment in all but one of the samples (Fig. 3a). On average, the mapping rates were 8.26, 9.93, and 5.13% higher than the CJ2010, CJ2015, and CJ2017 assemblies respectively. Also, Human Gencode (Release 29) transcripts, mapped more to the CJ2019 genome than the previous versions. When BAC-end data were mapped, against the current chromosomal genomes (CJ2010 and CJ2015) at NCBI, the difference in mapping rates was more than 12% (Fig. 3b). Compared to the CJ2017 assembly, the concordant mapping rate of BAC-end data was increased by 6.30%. The increase in mapping rates is further proof that the genome has been improved significantly.

### Possible structural errors

**Fig. 4:**
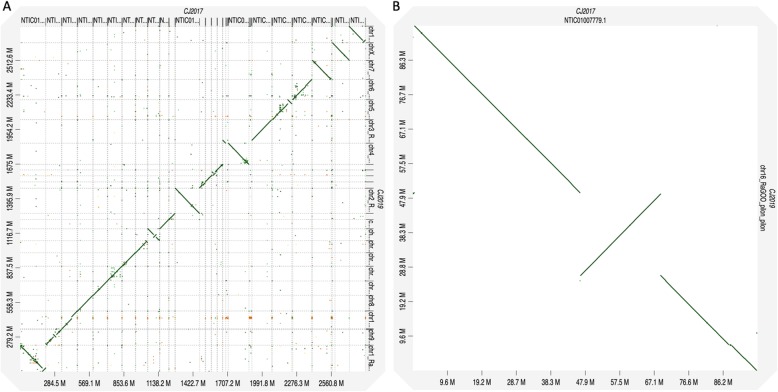
Dot plot of the alignment between CJ2017 and CJ2019. **a**) Whole genome view, **b**) Focused view of chromosome 16. The X-axis represents CJ2017 scaffolds and Y-axis represent CJ2019 scaffolds. A diagonal straight line indicates synteny among the genomes

CJ2017 and CJ2019 genome assemblies were aligned against each other with minimap2 [28] and visualized using dot plots generated by d-genies [29]. Numerous small and large inversions were observed between the two assemblies. Hi-C scaffolding can erroneously introduce inversions in short contigs [30], and this can be attributed to the small inversions observed in the dot plots (Fig. 4a). However, larger inversions, such as that observed in chromosome 16 (Fig. 4b), could be actual misassembled structural errors. The mapping of long-range paired BAC-end reads did not support the chromosome 16 inversion of CJ2019 genome, indirectly hinting that the make-up at this particular location is more accurate in CJ2017 genome. It has to be noted that the original marmoset genome assembly was constructed with human genome as a guiding factor, and hence some parts are effectively humanized and could be actually structural errors. A new common marmoset genome, as a part of the Vertebrate Genome Project, is under development, which includes 55.69 x coverage of 10x genomics data, 105.81 x coverage of Arima Hi-C data, 154.52 x coverage of BioNano optical map data, along with PacBio and Illumina sequence reads [31]. Although vast improvements in terms of contiguity, sequence gaps, and mapping rates of various genomic elements could be observed in the CJ2019 assembly, structural errors could potentially remain which would need the combination of the above data to effectively resolve them.

## Conclusions

The high-quality genome constructed as part of this manuscript has shown vast improvements in terms of contiguity, gaps, genomic features, and mapping rates of sequence data and will be widely useful for researchers involved in the field of primate genomics.

## Methods

### Sample preparation and sequencing

The liver sample (Animal I2075) from the Central Institute for Experimental Animals (CIEA), Japan, was originally used to improve the Marmoset genome using Illumina [10], with ample part of the animal’s samples retained for future use. Briefly, an 8-year, 4-month-old male marmoset liver was used for DNA extraction using the phenol-chloroform-isoamyl alcohol extraction method. The genomic DNA for long-read sequencing in this study was extracted from the kidney of the same animal described above. The genomic DNA was extracted using QIAGEN Genomic-tip 500/G (Qiagen, Hilden, Germany) according to the manufacturer’s instruction. To avoid blood contamination, bloodletting was performed completely at the dissection. The genomic sample was sequenced using PacBio RSII sequencer on 93 SMRT cells. In addition, RNA sequencing was also employed to improve the prediction of gene models. Two male marmosets (Animals: I5998 and I6289) that were 2 years old and obtained from CLEA Japan Inc. were deeply anaesthetized with isoflurane and euthanized by exsanguination from the femoral artery. Twelve tissue samples from the brain (Table S5) were collected and immediately frozen using liquid nitrogen and were rapidly broken down in solution D using a polytron homogenizer, before extracting the total RNA by the acid guanidinium thiocyanate-phenol-chloroform method. The quality and the concentration of the sample were measured using the 2100 Bioanalyzer instrument with the Agilent RNA 6000 Nano kit. The cDNA sequencing libraries were constructed with 1000 nanograms of total RNA using the TruSeq RNA Sample Prep Kit (Illumina), following the instructions in the TruSeq RNA Sample Preparation V2 Guide (Illumina). MiSeq Reagent Kit v3 (600-cycles) was used for sequencing the RNA samples (2 * 150 bp PE reads) with the MiSeq sequencer.

### De novo assembly and pseudo-chromosome construction

The sequenced reads were input to several assembly tools as recommended in the benchmark article [32], including Canu [33], SMARTdenovo [34], wtdbg [35], miniasm [36], Flye [37], Falcon [38], and MECAT [39], as part of the assembly workflow (Fig. 1). MECAT aborted with a segmentation fault, while Falcon produced relatively shorter contigs, and both were subsequently left out from the further analysis. Quiver [40] was executed in two iterative rounds to polish all the assemblies. In the case of wtdbg and miniasm assemblies, an additional round of quiver was performed owing to their relatively high error rates in the consensus sequences. RaGOO [41] was used to construct pseudo-chromosomes from the assembled contigs by using the previous marmoset genome [10], as a reference. To fill the gaps, a hybrid assembly was constructed by mapping, using minimap2, the flanking regions of sequence gaps in the SMARTdenovo assembly against the contiguous regions of the other assemblies, and replacing the gaps with nucleotide bps. Two rounds of consensus polishing by Pilon [42], using Illumina data from the same sample [10], was also executed to polish the assemblies further.

### Genome annotation

Repeat content was assessed using RepeatModeler and RepeatMasker [43]. The repeats, in the contiguous sequences of the assembly, were first identified by RepeatModeler to construct repeat family libraries, which were in turn used by RepeatMasker to annotate and mask the repetitive regions of the assembled pseudo-chromosomes. For gene annotation, a random set of 1000 multi-exon genes were obtained from Ensembl (Release version 95) database’s common marmoset gene annotation to train gene models using Augustus [44]. In addition, a combination of ab-initio based, homology based, and transcriptome-based strategies were applied to predict and update the predicted genes. In the homology based approach, protein sequences were collected from a) the recently assembled Chimpanzee and Orangutan genomes [13], from NCBI, b) NCBI’s NR protein database for the Gorilla genome, and c) the Gencode (Release 29) database for the Human genome, and were aligned against the assembled genome using funannotate [45], which in-turn uses diamond [46], and exonerate [47]. Parallelly, Marmoset ESTs downloaded from NCBI were input to PASA [48], to model gene structures from the EST alignments. These EST and protein alignments were provided as hints for the Augustus ab-initio gene prediction program with the trained gene model as the species parameter on the assembled genome, which was earlier soft-masked for repeats using RepeatMasker. Later, RNAseq reads from the 12 different brain tissues (Table S5), as well as marmoset samples from the study, SRP051959 [49], were de novo assembled into transcripts using Trinity [50]. PASA was used once again, in conjunction with the Trinity transcripts, to update the UTR and alternate splicing information of the predicted genes.

### Evaluation of the assembly

BUSCO was used to evaluate the completeness of the evolutionarily conserved genes in the assemblies. To further evaluate the quality of the de novo assembly, different sets of DNA and RNA data were aligned against the assembled genomes. RNAseq reads, Human Gencode transcripts, and BAC-end data, were aligned against the genome assemblies using STAR [51], GMAP [52], and bowtie2 [53] aligners respectively. Minimap2 [28] was used to align the whole genome assemblies against each other, and d-geneis [29] was used to obtain and visualize dot plots from the alignments.

## Supplementary information

**Additional file 1.** Parameters used for the tools.

**Additional file 2: Figure S1.** Histogram of the sequenced read lengths.

**Additional file 3: Figure S2.** Comparison of the N50 and N75 values across all the assembled genomes.

**Additional file 4: Table S1.** Assembly statistics of the marmoset genome from different assemblers.

**Additional file 5: Table S2.** Pseudo-chromosome statistics of the marmoset genome assemblies from different assemblers.

**Additional file 6: Table S3.** Repeat statistics of the final marmoset genome assembly.

**Additional file 7: Table S4.** Summary of the marmoset genome assemblies in the NCBI Assembly database.

**Additional file 8: Table S5.** RNAseq of brain tissue samples.

## Data Availability

All data has been submitted to the DNA DataBank of Japan (DDBJ). The Bioproject, the Biosample, and the DDBJ read archive accession numbers for the genome data are PRJDB8242, SAMD00169834, and DRA008324 respectively. The genome assembly has the accession numbers: BJKT01000001–BJKT01000065. The RNAseq data has accession numbers: DRA008173, DRA006301, and DRA008220.

## Abbreviations

SMRT: Single Molecule Real Time
RNA: RiboNucleic Acid
LINE: Long Interspersed Nuclear Elements
SINE: Short Interspersed Nuclear Elements
LTR: Long Terminal Repeats
NCBI: National Center for Biotechnology Information
NR: Non Redundant database
CDS: Coding sequence
EST: Expressed Sequence Tags
bp: basepair
kbp: kilo basepair
Mb: mega basepair
Gb: Giga basepair
BAC-end: Bacterial Artificial Clone end
