# Supplementary material for: An improved de novo genome assembly of the common marmoset genome yields improved contiguity and increased mapping rates of sequence data
Source: BMC Genomics. 2020 Apr 2;21(Suppl 3):243. doi: 10.1186/s12864-020-6657-2 (PMC7114785; doi:10.1186/s12864-020-6657-2)
Supplement: Supplementary file 1 — Additional file 1. Parameters used for the tools. [file 12864_2020_6657_MOESM1_ESM.pdf]

Parameters used for the tools

Canu version 1.4 (r8079 00bed61d95d4c54522170099a5e8c366f52997c6):
Commands and parameters
canu -assemble genomeSize=2.26G

Wtdbg version 1.2.8 (r1 20180529):
Commands and parameters

```
wtdbg: -i reads -fo m --tidy-reads 5000 -k 0 -p 21 -S 4 --rescue-low-cov-edges
wtdbg-cns: -i m.ctg.lay -fo m.ctg.lay.fa -c 0
kbm: -d m.ctg.lay.fa -i reads -k 0 -p 21 -S 4 -O 0 | best_kbm_hit.pl | awk '{print      \\
$6"\t"$9"\t"$10"\t"$1"\t"$2"\t"$4"\t"$5}' >m.map
map2dbgcns: m.ctg.lay.fa reads m.map >m.map.lay
wtdbg-cns: -I m.map.lay -fo m.map.fa -k 13 -c 3
```

Flye version 2.3.4:
Commands and parameters

```
Flye: --pacbio-raw reads -g 2.9g
```

SMARTdenovo (git commit 3d9c22e25bdf4caf6c08ea1acb41ee58e52f61a8):
Commands and parameters: default (with consensus generation)

Minimap (git commit 1cd6ae3bc7c7a6f9e7c03c0b7a93a12647bba244) and miniasm (git commit 17d5bd12290e0e8a48a5df5afaeaef4d171aa133):
Commands and parameters

```
Minimap: -Sw 5 -L 100 -m 0 reads reads >m.paf
Miniasm: -f reads m.paf >m.gfa
```

MECAT (git commit 3898797d5d0ead78b14af65089f6be32263ca103):
Commands and parameters

```
mecat2pw: -j 0 -d reads -o m.pm.can -w.
mecat2cns: -i 0 m.pm.can reads corrected.fastq
extract_sequences: corrected.fastq corrected_25x.fastq 2900000000 25
mecat2canu: -assemble -p m -d m genomeSize=2.9G ErrorRate=0.02
maxMemory=350 maxThreads=30 useGrid=0 Overlapper=mecat2asmpw -pacbio-corrected
corrected_25x.fastq.fasta
```

FALCON-integrate (git commit b22b63d6811b2be612db608b0fb62e6843f6c4c8):
Commands and parameters

```
length_cutoff_pr: 10000
length_cutoff: 10000
pa_HPCdaligner_option:  -v -B24 -t16 -e.70 -l1000 -s500
ovlp_HPCdaligner_option: -v -B24 -t32 -h60 -e.96 -l500 -s500
pa_DBsplit_option: -x500 -s400
ovlp_DBsplit_option: -x500 -s400
falcon_sense_option: --output_multi --min_idt 0.70 --min_cov 4 --max_n_read 200 --
n_core 6
```

overlap_filtering_setting: --max_diff 100 --max_cov 100 --min_cov 4 --bestn 10 --n_core 24

Quiver (from smrtanalysis-patch_2.3.0.140936.p5):
Commands and parameters
minLength: 500
minSubReadLength: 500
readScore: 0.80
maxHits: 10
maxDivergence: 30
minAnchorSize: 12
placeRepeatsRandomly: True
pbalign_opts: --seed=1 --minAccuracy=0.80 --minLength=500 --algorithmOptions='-useQuality'

RaGOO version 1.01:
ragoo: -b -m minimap2 assembly.fasta reference.fasta

Bwa version 0.7.12 (for alignment of Illumina reads before pilon):
Commands and parameters
mem: -I {500,700,2571,2731,7583,19833,39833} (for every PE and MP dataset)
Bwa mem was used for single end reads also. The sam files were sorted and indexed using samtools.

Pilon version 1.23:
Commands and parameters
pilon: --fix bases --vcf --changes –diploid
Pilon was executed with all PE and MP libraries for individual chromosomes.

Repeat Masking:
RepeatModeler version 1.0.11:
Commands and parameters
BuildDatabase: -name a -engine ncbi contigs.fa
RepeatModeler: -engine ncbi -database a >a.out

RepeatMasker version 4.0.8:
Commands and parameters
RepeatMasker: -dir A -xsmall -lib consensi.fa.classified assembly.fa
