# Supplementary figures and images for: An improved de novo genome assembly of the common marmoset genome yields improved contiguity and increased mapping rates of sequence data
Source: BMC Genomics. 2020 Apr 2;21(Suppl 3):243. doi: 10.1186/s12864-020-6657-2 (PMC7114785; doi:10.1186/s12864-020-6657-2)

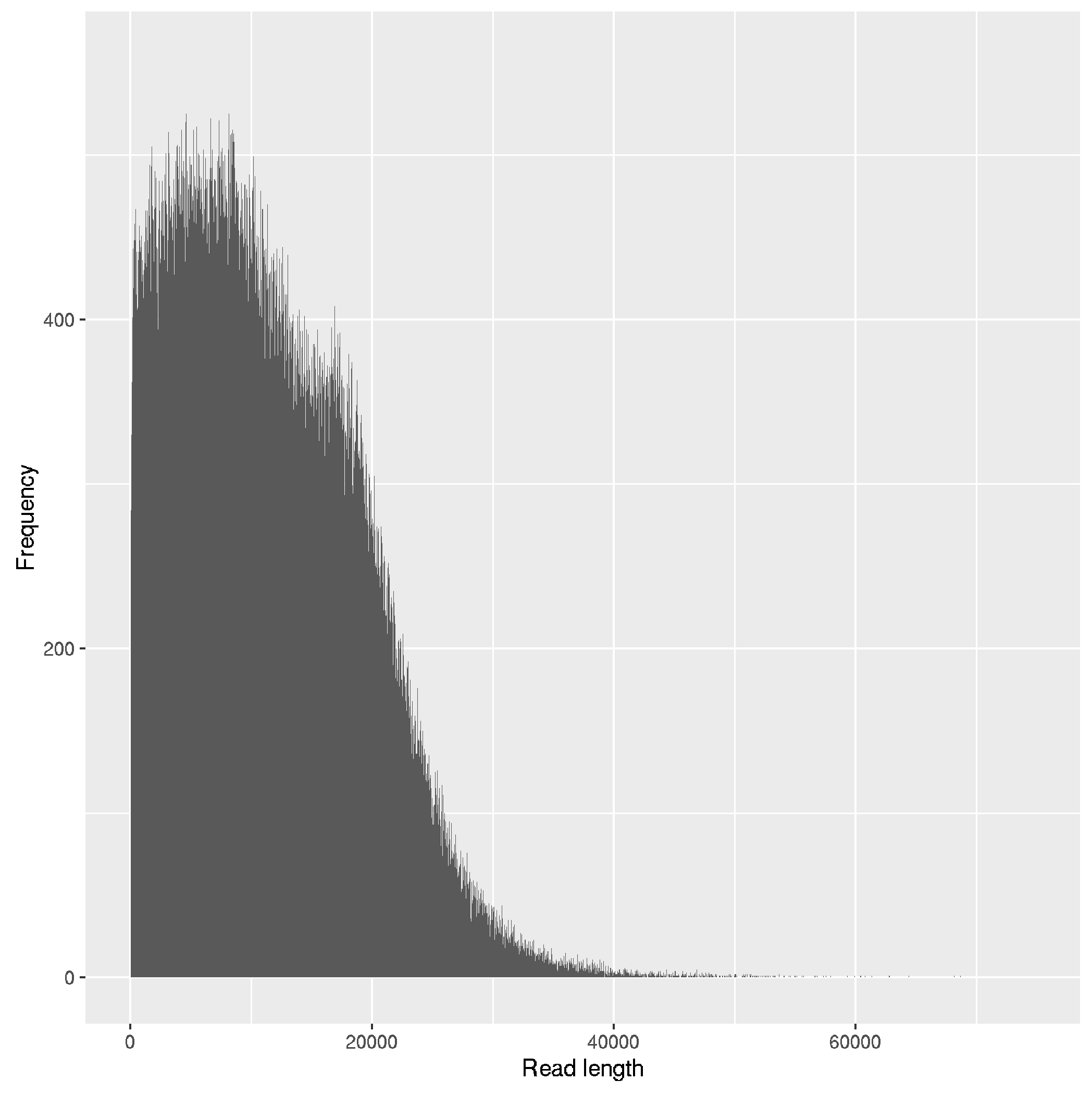

Supplement: Supplementary file 2 — Additional file 2: Figure S1. Histogram of the sequenced read lengths. [file 12864_2020_6657_MOESM2_ESM.png]

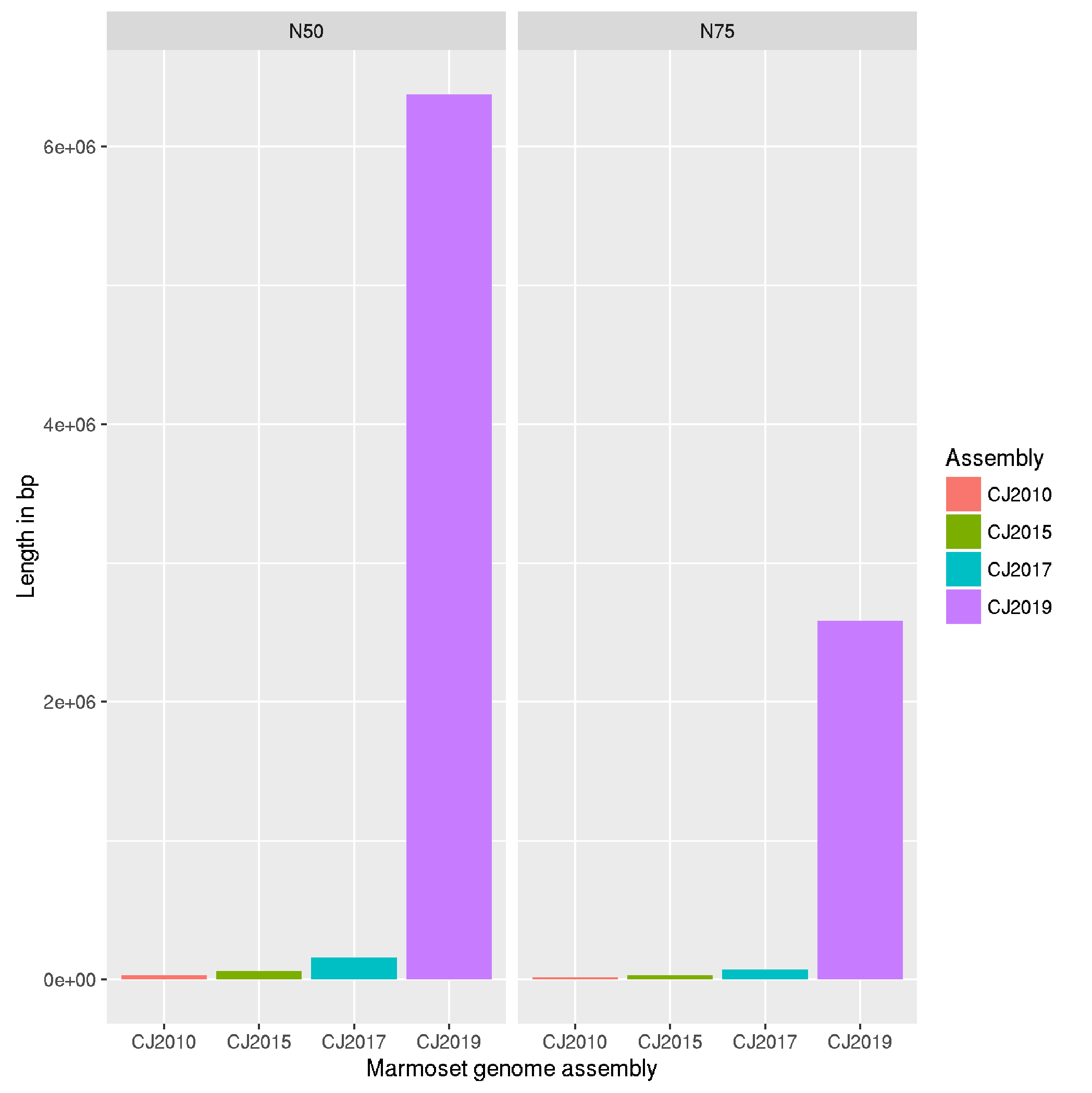

Supplement: Supplementary file 3 — Additional file 3: Figure S2. Comparison of the N50 and N75 values across all the assembled genomes. [file 12864_2020_6657_MOESM3_ESM.png]
